# FH ALERT: efficacy of a novel approach to identify patients with familial hypercholesterolemia

**DOI:** 10.1038/s41598-021-99961-y

**Published:** 2021-10-14

**Authors:** Felix Fath, Andreas Bengeser, Mathias Barresi, Priska Binner, Stefanie Schwab, Kausik K. Ray, Bernhard K. Krämer, Uwe Fraass, Winfried März

**Affiliations:** 1 SYNLAB Holding Germany GmbH, SYNLAB Academy, Mannheim, Germany; 2 Vth Department of Medicine (Nephrology, Hypertensiology, Rheumatology, Endocrinology, Diabetology), Medical Faculty Mannheim, University of Heidelberg, Mannheim, Germany; 3 SYNLAB Holding Germany GmbH, Augsburg, Germany; 4 SYNLAB MCC Human Genetics Mannheim GmbH, Mannheim, Germany; 5 Imperial Centre for Cardiovascular Disease Prevention, Department of Primary Care and Public Health, Imperial College London, London, UK; 6 European Center for Angioscience ECAS, Medical Faculty Mannheim, University of Heidelberg, Mannheim, Germany; 7 Amgen GmbH, Munich, Germany; 8 Clinical Institute of Medical and Chemical Laboratory Diagnostics Medical, University of Graz, Graz, Austria; 9 D A CH Society Prevention of Cardiovascular Diseases e.V., Hamburg, Germany

**Keywords:** Risk factors, Dyslipidaemias

## Abstract

Diagnosis rates of familial hypercholesterolemia (FH) remain low. We implemented FH ALERT to assess whether alerting physicians for the possibility of FH impacted additional diagnostic activity. The study was conducted from SYNLAB laboratory Weiden (Bavaria). Beyond common reporting of LDL-C or TC, 1411 physicians covering approximately a population of 1.5 million people were eligible to receive an alert letter (AL) including information on FH, if laboratory results exceeded thresholds as follows: adults LDL-C ≥ 190–250 mg/dl (to convert into mmol/l multiply with 0.0259), TC ≥ 250 to ≤ 310 mg/dl (probable suspicion); LDL-C > 250 mg/dl and TC > 310 mg/dl (strong suspicion). Persons below 18 years were alerted for LDL-C  140 mg/dl and TC ≥ 200 mg/dl (strong suspicion). Patients above 60 years were excluded. Our readouts were characteristics of involved physicians, rate of ALs issued, acceptance, and subsequent diagnostic activity. Physicians were mainly general practitioners in ambulatory care. 75% of the ordered tests were for TC, 25% for LDL-C. We issued 3512 ALs (~ 5% of tests) triggered by 2846 patients. 86% of eligible physicians stayed with the initiative, 32.7% were alerted, and 70% were positive upon call-center survey. We registered 101 new visitors of www.fhscore.eu and sent out 93 kits for genetics. Thereof, 26 were returned and 5 patients were positive for FH. Physicians were in general open to our approach. Although genetic testing was taken up with caution, this 3-months pilot examination resulted in a greater rate of patients with FH diagnosed than previous screening projects. Further education on FH in primary care is required to improve FH detection in the community.

## Introduction

Elevated low-density lipoprotein cholesterol (LDL-C) is among the most important causes of atherosclerosis^1^. Genetic epidemiological and interventional studies have shown a continuous relationship between LDL cholesterol and the incidence of atherosclerosis-related vascular events^1^.

Familial hypercholesterolemia (FH) is a genetic disorder of LDL-C metabolism, mainly caused by mutations in the genes encoding the LDL receptor (*LDLR*), apolipoprotein B100 (*APOB*) or proprotein convertase subtilisin/kexin type 9 (*PCSK9*). FH is inherited in an autosomal dominant manner, meaning that 50% of the descendants of a heterozygote parent are affected^2^. FH is the most frequent autosomal dominant genetic disease encountered in adults with a global prevalence estimated at between 1:200 and 1:300 and more common among populations with founder effects^3^. FH is characterized by pronounced elevations of plasma LDL-C as early as in childhood and premature onset of coronary heart disease^2^. Roughly 2% of individuals with an LDL-C above 190 mg/dL (to convert into mmol/l multiply with 0.0259) suffer from FH^4^. Homozygous FH (HoFH) frequently results in cardiovascular events in the first decade of life, and left untreated those with HoFH may die before the age of 20 years^5^. In heterozygous (HeFH) patients the additional risk of cardiovascular events is 50% in men up to the age of 50 and 30% in women up to the age of 60^6^.

Despite all available data detection remains low. For instance the prevalence rate of HeFH is approximately 1:300^7^, which entails more than 250,000 patients in Germany^6^, yet the current diagnosis rate is estimated to be between 1 and 2%^3^. Unidentified and untreated patients on average lose 15–21 years of life^8^. Registry data from the Netherlands have shown that an early screening strategy together with effective and early use of statin therapy to reduce LDL-C can reduce the cardiovascular risk associated with FH similar to that of the general population^9^. Thus, it is crucial that patients are identified early in life. Although, there are several clinical algorithms which help to identify patients with FH^10–12^, they are underutilised in clinical practice. Moreover, these algorithms can be used to increase the pre-test probability of a positive genetic test^13^. The clinical value of genetic diagnostics of FH has been underscored by guidelines and expert consensus in the United States^14,15^, Europe^16,17^, and Germany^18^, yet, utilization of genetic testing remains poor. To facilitate the diagnosis of FH and given that this condition is dependent upon ascertainment of an elevated LDL-C measurement, we designed a pilot study (FH ALERT) to serve as the interface between a clinical laboratory receiving blood samples for lipid measurements and the ambulatory outpatient care, to assess whether a decision support protocol was feasible, acceptable, and improved detection rates compared to historical records. Here we report the results of this pilot study to evaluate feasibility and acceptance of this approach in a German outpatient care environment.

## Materials and methods

### Setting

We included all ambulatory care physicians (general practitioners and specialists across all disciplines) referring their laboratory samples to the SYNLAB Medical Care Center (MCC) Weiden GmbH which is located in Bavaria, Germany. We chose MCC Weiden GmbH because it is one of the biggest SYNLAB core laboratories in Germany. All patients aged 60 and below visiting participating physicians who were being evaluated for elevated LDL-C or total cholesterol (TC) measurements were eligible. The study was conducted between March 15, 2018 and June 15, 2018 and approved by the Ethics Committee II of the Medical Faculty Mannheim, Heidelberg University, Theodor-Kutzer-Ufer 1-3, 68167 Mannheim (reference number 2018-849R-MA). As this was a retrospective evaluation of findings generated during regular patient care and no study specific procedures were conducted, the above-mentioned ethics committee did not consider it necessary to obtain individual informed consents. All methods were carried out in accordance with relevant guidelines and regulations.

### Procedures

**Figure 1:**
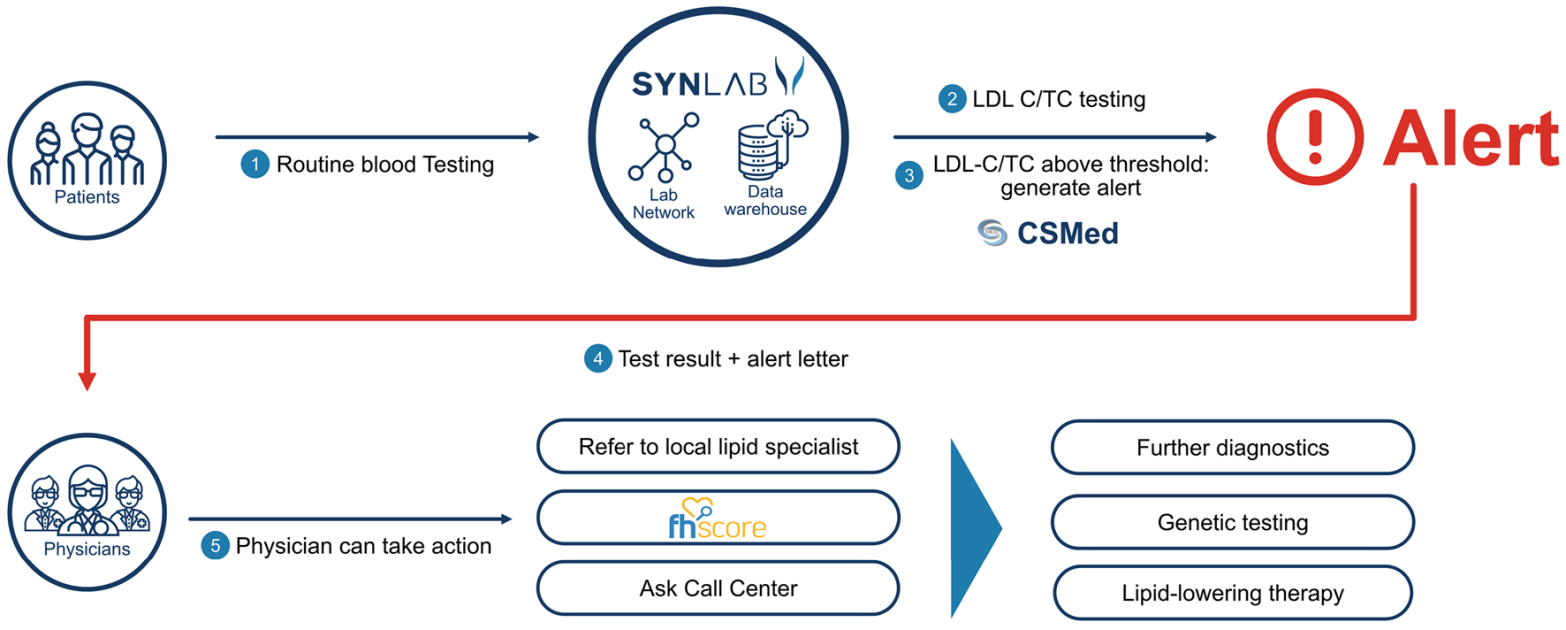
Schematic representation of procedures of FH ALERT. Alerting letters (ALs) were issued to treating physicians once total cholesterol (TC) or LDL-C scored above predefined threshold values. In addition to TC or LDL-C the ALs provided information about FH and further diagnostic options including genetic testing.

Beyond the regular laboratory results, participating physicians received an alert letter (AL) once TC or LDL-C levels exceeded predefined threshold values (Fig. 1). The ALs also included scientific information about FH and the further diagnostic options including genetic testing.

We used six different threshold values to trigger an AL (Table 1). We distinguished among two categories with different wording, namely “suspicion of FH” in adults belonging to the LDL-C threshold category I or TC threshold category I as well as “strong suspicion of FH” in children and in adults belonging to LDL-C threshold II and/or TC threshold II (Table 1). LDL-C was used as the primary criterion to trigger reports, TC was used in cases in which LDL-C was not available. We recommended the Dutch Lipid Clinic Network (DLCN) score to estimate the pre-test probability of a positive genetic test, with a test score of ≥ 6 indicating for genetic testing.

**Table 1:** Overview of defined thresholds triggering alert letters.

	Children (< 18 years) (mg/dl)	Suspicion of FH	Adults (≥ 18 ≤ 60 years) (mg/dl)	Suspicion of FH
LDL C threshold I	≥ 140	Strong	≥ 190	Probable
LDL C threshold II			> 250	Strong
TC threshold I *(if LDL C is not available)*	≥ 200	Strong	≥ 250	Probable
TC threshold II *(if LDL C is not available)*			> 310	Strong

Beside the thresholds, alert categories and associated severity levels are illustrated.

Approximately two weeks before initiation, we announced FH ALERT by an initiation letter to all physicians regularly referring tests to the SYNLAB MCC Weiden GmbH. This document described background and aims, design and procedures of the project. The initiation letter and each subsequent AL informed the participating physician that they had the choice to opt-out from the project at any time.

The software extracting TC or LDL-C from the laboratory information system to produce the ALs was written by CSMed GmbH, Darmstadt, Germany. We established a call center which was specifically instructed and available to participating physicians to provide further information on the diagnoses of FH if requested (inbound calls). The call center was operated by IMS Health GmbH & Co. OHG, Bensheim, Germany. The ALs usually arrived the day following the regular report. They included in depth information on the diagnosis of FH, advice how to use the DLCN score^12^, how to initiate genetic testing, and named lipid specialists in the surroundings of the patients’ residential address. Any medical decisions regarding further diagnostic and therapeutic measures were completely left to the discretion of the responsible physicians (Fig. 1).

Physicians who received at least one AL were contacted pro-actively by the call center and asked to assess the FH Alert project using a standardized questionnaire. Physicians were also encouraged to express their criticism and to propose improvements of the procedures.

### Genetic testing

Current guidelines and expert opinion suggest that the diagnosis of FH is established by genetic testing if accessible^14–18^. To achieve seamless access to genetic testing for FH, we offered physicians the facilities at the SYNLAB Center of Human Genetics in Mannheim, Germany. Interested physicians were provided with kits (including request and consent forms and material for sampling) upon request. We monitored the number of genetic tests requested during the FH ALERT project until 6.5 months after the conclusion of the project on June 15, 2018. This accounted for delays in ordering genetic tests due to waiting for the patients’ next appointments, education of patients or receiving their informed consent.

Patients’ DNA was isolated from a blood sample and then analyzed by next generation sequencing. Following library preparation with TruSight Rapid Capture technology and bridge amplification, the sample was sequenced on an Illumina NextSeq 500 instrument. Coverage of the ROI was > 98%, reading depth a minimum of 100-fold. We examined the following loci: *LDLR, APOB, PCSK9, LDLRAP, SORT1, NPC1L1, STAP1, APOE, ABCG5, ABCG8, DHCR24, LIPA, CYP27A1*, and *DHCR7*. In addition, the *LDLR* gene was also evaluated for copy number variation by multiplex ligation-dependent probe amplification. Variants were classified according to current American College of Medical Genetics and Genomics (ACMG) guidelines^19^. Results were classified as inconspicuous, polygenic hypercholesterolemia and classically monogenic.

### Outcomes

This study used the following readouts: characteristics of the physicians involved, number of ALs issued in comparison to the number of TC and/or LDL-C, acceptance by physicians, further diagnostic activity including genetic testing.

### Statistics

Statistics is descriptive. Beyond the algorithm to generate alerting reports, CSMed GmbH also provided a documentation tool and a customized statistics module to evaluate FH ALERT. All calculations were performed with Microsoft^®^ Excel^®^ 2016 (Version: MSO (16.0.9126.2282) 32-Bit). The frequency of the use of the FH score online tool was tracked with Google Analytics.

## Results

### Characteristics of the physicians involved

**Figure 2:**
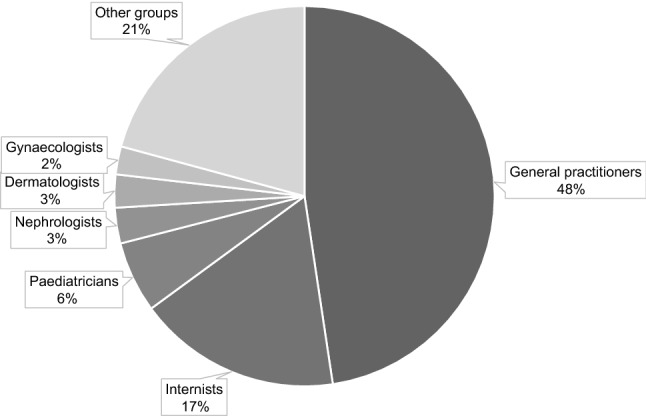
Distribution of Alerting letters (ALs) to specialist groups. Most ALs were issued to general practitioners, followed by internists while paediatricians, nephrologists, dermatologists and gynaecologists contributed marginally.

During the project period 1411 physicians/institutions ordered at least one analysis of any parameter from the SYNLAB MCC Weiden. They were mainly in ambulatory care (general practitioner or medical specialist), but also included occupational health departments of companies or health care institutions. Most letters were issued to general practitioners, followed by internists while paediatricians, nephrologists, dermatologists and gynaecologists contributed marginally (Fig. 2).

### Alert letters generated

During the project period of 3 months, 193,715 samples were analyzed by the SYNLAB MCC Weiden GmbH, and 75,431 TC and/or LDL-C examinations were performed. The 75,431 requests for cholesterol/LDL cholesterol originated from 60,812 patients. The difference is due to the fact that 7479 patients were tested twice, 2679 were tested three times, 274 were tested four times, and 145 were tested five times or more. Hence, the reference population included 60,812 persons. During our observation period, TC and/or LDL-C were therewith the fourth most frequent tests ordered, ranking just behind alanine aminotransferase (ALAT), aspartate aminotransferase (ASAT) and thyroid stimulating hormone (TSH). Overall, 3512 (4.7%) ALs were issued. These alerts were triggered by 2846 patients. In 885 (of 3512) cases (25.2% of 3512) the ALs were triggered by LDL-C with or without TC, whereas in 2627 cases (74.8% of 3512) TC only triggered the ALs. Amongst those, approximately 95% fell into to category I. 154 (4.4%) of all ALs were generated patients below 18 years of age. In category II, all 181 ALs were for adults. The average age of alerted persons was 48 years in total, 50 years in women and 47 years in men (Table 2).

**Table 2:** FH ALERT’s pilot initiative key results.

Period of time: 12 weeks	Absolute values
Cholesterol examinations	75,431
Number of patients in whom examinations were performed	60,812
Patients tested twice	7479
Patients tested three times	2679
Patients tested four times	274
Patients tested five times or more	145
Alert letters, n (%)	3512 (4.66)
Number of patients triggering Alert letters	2846
**Distribution of alerts**	
Category I	
Adults with probable suspicion	3177
Children with strong suspicion	154
Sum, n (%)	3331 (94.85)
Thereof LDL C, n (%)	838 (25.16)
Thereof TC, n (%)	2493 (74.84)
Category II	
Adults with strong suspicion, n (%)	181 (5.15)
Thereof LDL C, n (%)	47 (25.97)
Thereof TC, n (%)	134 (74.03)
Age	
Patients < 18 years, n (%)	154 (4.38)
Patients ≥ 18 ≤ 60 years, n (%)	3,358 (95.62)
Average age (total/ female/male, years)	48/50/47
**Acceptance and feedback**	
Total number of clients of MCC Weiden GmbH, n (%)	1411 (100)
Clients with Opt-out, n (%)	200 (14.17)
Alerted clients	
Clients with alert letters, n (%)	462 (32.74)
Alert letters per client (total/alerted clients)	2.49/7.60
≥ 10 alert letters (Opt-in, cum.), n (%)	121 (26.19)
≥ 5 ≤ 9 alert letters (Opt-in, cum.), n (%)	48 (10.39)
≥ 3 ≤ 4 alert letters (Opt-in, cum.), n (%)	37 (8.01)
Positive reaction by caller, n (%)	370 (69.94)
Negative reaction by caller, n (%)	38 (7.18)
Undifferentiated reaction by caller, n (%)	121 (22.87)
FH score	
New visitors/recurring visitors	101/31
Completed questionnaires	88
Questionnaires > 3	45
**Genetic testing**	
Requested genetic test kits	93
Returned genetic test kits, n (%)	26 (27.96%)

Data describes general information, distribution of alerts, acceptance, feedback, and genetic testing.

On average, there were 2.49 ALs per physician, when calculated based on 1411 (total participating physicians) and 7.6 ALs per physician in relation to physicians who received at least one AL. 462 of participating physicians—predominantly general practitioners and internists—were alerted and received at least one AL (32,74%). 37 medium frequently alerted physicians received 3–4 ALs (8.01% of alerted physicians), 48 frequently alerted physicians 5–9 ALs (10.39% of alerted physicians) and 121 high frequently alerted physicians were alerted ten times or more (26.19% of alerted physicians).

### Acceptance

Only 200 physicians chose to opt out from the project and 1211 physicians participated for all 3 months, corresponding to an opt-out rate of 14.2%; 32.74% of all physicians received at least one AL (Table 2).

In the survey conducted by the call center, FH ALERT was evaluated positively by approximately 70% of respondents, 23% did not finally know how to classify and 7% had a negative opinion about FH ALERT. The most frequent criticism arose regarding to the amount of paperwork caused by ALs, followed by a lack of additional time to look through the ALs.

The FH score website was visited 101 times due to the FH ALERT project; 31 visitors recurred at least once, 88 FH score questionnaires were completed whereof 45 had a result of ≥ 3 (Table 2).

### Further diagnostic activity including genetic testing

During the FH ALERT project 93 sampling kits for genetic testing were sent out to 60 physicians and 26 were returned for analysis within the active period and 6.5 months beyond. Of these, ten samples were inconspicuous, three showed variants most likely not related to hypercholesterolemia, eight samples showed variants or combinations of variants which we classified as polygenic hypercholesterolemia, and five samples showed at least one variant that we deemed causal for familial hypercholesterolemia. Among these, three were heterozygous for *LDLR* mutations, one carrier of a PCSK9 mutation and one carrier of an APOB mutation (Table 3).

**Table 3:** Genetic findings in 26 samples obtained from FH ALERT.

Diagnosis	Gene	Variant	Zygosity	ACMG class	Remarks
**Variants most likely not relevant to hypercholesterolemia**					
	*CYP27A1*	c.1494G>C, p.(Lys498Asn)	Het	3	Recessive mutations of *CYP21A1* responsible for CTX, not described in the literature, in silico possibly damaging, but Lys498 poorly conserved and CTX patients have typically low to normal LDL C^20^
	*NPC1L1*	c.448C>T, p.(Leu150Phe)	Het	3	Leu150 poorly conserved, not contained in mutation databases, not described in the literature, in silico benign
	*NPC1L1*	C1496C>T, p.(Thr499Met)	Het		Thr499 poorly conserved, pathogenic according to HGMD, loss of function variant, reduced intestinal cholesterol absorption^21,22^
**Possibly relevant to hypercholesterolemia**					
Genetic HC	*APOE*	c.526C>T, p.(Arg176Cys)	APOE2/3		Type III HLP?
	*APOE*	c.388T>C, p.Cys130Arg	APOE3/4		LDL-C increased by apoE4^23^
	*ABCG5*	c.431T>C, p.(Val144Ala)	Het	3	Val144 strongly conserved, low frequency, located in the “P-loop containing nucleoside triphosphate domain” of ABCG8, in silico disease causing, no reports in the literature, increased cholesterol absorption?
	*DHCR24*	c.231 + 19G>C, p.(?)	Het	3	Intronic variant, in silico prediction of aberrant mRNA splicing, no reports in the literature
	*PCSK 9*	c.1327G>A, p.(Ala443Thr)	Het	3	Ala443 poorly conserved, no relationship to elevated LDL-C^24^
	*ABCG8*	c.712G>A, p.(Glu238Lys)	Het	3	Glu238 strongly conserved, located in the “P-loop containing nucleoside triphosphate domain” of *ABCG8*, in silico disease causing, loss of acceptor splice site of exon 6, no reports in the literature, increased cholesterol absorption?
	*APOE*	c.526C>T, p.(Arg176Cys)	APOE2/3		Type III HLP?
	*ABCG8*	c.1832G>A, p.(Arg611Lys)	Het	3	Arg611 poorly conserved, increased cholesterol absorption?
	*SORT1*	c.43C>T, p.(Pro15Ser)	Het	3	Pro155 poorly conserved, not contained in mutation databases, low allele frequency, in silico controversial
	*CYP27A1*	c.4391G>A, p.(Arg164Gin)	Het	3	Arg164 highly conserved, in silico controversial, low allele frequency, not described in the literature
	*APOE*	c.388T>C, p.Cys130Arg, c.137T>C, p.(Leu46Pro)	APOE3/4P		ApoE-Pittsburg, possibly associated with Alzheimer’s disease^25,26^, association with cholesterol elusive
	*ABCG8*	c.1083G>A, p.(Trp361*)	Het		Causes stop of the translation, causal in recessive sitosterolemia^27,28^, increased cholesterol absorption?
	*APOE*	c388T>C, p.(Cys130Arg)	APOE3/4		LDL-C increased by apoE4^23^
	*APOB*	c.3337G>C, p.(Asp1113His)	Het	3	Asp1113 poorly conserved, controversial in silico*,* p.Arg1164Thr in the vicinity considered causal in one publication^29^
	*NPC1L1*	Haplotype c.529G>A p.Val177Ile, c.661C>T p.(His221Tyr) c811_812delGCinsTT p.(Ala271Phe)	Het	3	c.529G>A and c.661C>T reported associated with high HDL-C and TC^30^
**Familial hypercholesterolemia**					
Het FH	*ABCG5*	c.1829A>C, p.(Glu610Ala)	Het	3	Low frequency, in silico disease causing, high cholesterol absorption?
	*APOB*	c.2630c>T, p.(Pro877Leu)	Het	3	Pro877 strongly conserved, in silico disease causing, no reports in the literature, binding deficient apo B-100?
	*PCSK9*	c.60_65*dup*GCTGCT, p.(Leu22_Leu23*dup*)	Het	3	Gain of function variant^31,32^
	*APOE*	c388T>C, p.(Cys130Arg)	APOE3/4		LDL-C increased by apoE4^23^
	*LDLR*	c.190 + 4_190 + 7*del,* p.?	Het	3	4 bp deletion within the donor splice of exon 4 likely likely to cause abberant splicing of the *LDLR*mRNA according to in silico prediction, individual base changes at c.190 + 4 and c.190 + 5 listed as pathogenic on HGMD, abberant splicing of these variants proven experimentally^33^
	*APOB*	c.6639_6641*del,* p.(Asp2213*del)*	Het	3	Variant previously detected in a patient with FH, causality still controversial^29,34^
	*APOB*	c.9242G>A, p.(Ser3081Asn)	Het	3	Significance of the variant unclear^35^
	*LDLR*	c.798T>A, p.(Asp266Glu)	Het		Asp266 highly conserved, disease causing according to HGMD and Clinvar, causal according to the literature^36,37^
	*LDLR*	c.10_*delins*CGGGGGCTGGAAATTGCGCTGGACCGTCGCC, c.10_*delins*31 p.(Trp4_Ala13*delins*ArgGlyLeuGlu IleAlaLeuAspArgArg), p.(Trp4_Ala13*delins*10)	Het	4	Exchange of 10 amino acids in exon 1, in silico pathogenic, not described in the literature nor in databases, multiple base changes have been described as pathogenic at position c.28; they replace Trp10 (which is affected by the current mutation as well) by other amino acids^36,38^
	*APOE*	c388T>C, p.(Cys130Arg)	APOE3/4		LDL-C increased by apoE4^23^

Subdivided according to the categories *variants most likely not relevant to hypercholesterolemia*, *possibly relevant to hypercholesterolemia* and *familial hypercholesterolemia* all findings are described by diagnosis, gene, variant, zygosity and ACMG (American College of Medical Genetics and Genomics) class as well as associated remarks in addition.

*ACMG* American College of Medical Genetics and Genomics, *Het* heterozygous, *CTX* cerebrotendinous xanrhomartosis,* HGMD* Human Gene Mutation Database, *HLP* hyperlipoproteinemia.

## Discussion

We successfully established a novel channel of information exchange between the clinical laboratory and requesting physicians. We found that this approach was generally welcome to the majority of the medical community participating. We also observed that this translated into further diagnostic steps as they are recommended by international and national expert opinion or guidelines^14–17^.

Even though TC and LDL-C are amongst the most frequently ordered tests in ambulatory care, the diagnosis rate of FH remains low in Germany, and in many other countries^3^. We started with the hypothesis that physicians commonly do not pay attention to extreme TC or LDL-C results included in conventional laboratory reports and hardly consider the diagnosis of FH, and if so, consecutive action or further diagnostic steps rarely follow. To enhance awareness for pathologic lipid parameters, we decided to specifically alert physicians once TC or LDL-C exceeded threshold values at which the probability of FH is deemed to be high.

### Alert letters generated

Approximately 5% of all the samples tested triggered an alert. This confirms that in the population studied, our thresholds defined a subgroup of samples beyond the 95^th^ percentile of either LDL-C or TC. Most of these samples fell in threshold category I (LDL-C 190–250, TC 250–310 mg/dl in adults or LDL-C ≥ 140 mg/dl, TC ≥ 200 mg/dl in children), only 5% fell in category II (LDL-C > 250 mg/dl or TC > 310 mg/dl). In line with this, the proportion of patients triggering an alert (2846 out of 60,812) was approximately 4.7% of persons tested at least once.

Only one quarter of the ALs was triggered by LDL-C with or without assessment of TC, the remaining ones by TC only. This stands in contrast to previous and current guidelines which define LDL-C as the primary treatment goal^16,39–41^ and demonstrates that further efforts are warranted to educate physicians to place emphasis on LDL-C instead of TC.

Of the participating physicians, who were predominantly general practitioners and internists, 32.74% received at least one report. This suggests that a small proportion of physicians include cholesterol and/or LDL-C testing into their standard practice of care. The majority of physicians may thus waive for screening for high cholesterol and/or to conduct state of the art risk assessment as it would comply with guidelines^16,17,39–41^. However, at least theoretically there may be physicians caring for patients with well controlled LDL levels or not being at elevated risk according to previous screening. As a consequence of the clustering of ALs to one third of the physicians only it may be worth limiting FH ALERT specifically to general practitioners and internists who regularly order TC and/or LDL-C in the future. In Germany, nephrologists and dialysis centres often provide care for patients with lipid disorders. However, they did not substantially contribute to the total number of ALs, on the one hand because they are small in number, on the other hand because their lipid patients may be well controlled (Fig. 2). Only few ALs originated from paediatricians, indicating that TC and/or LDL cholesterol are infrequently measured in patients < 18 years and that opportunities to identify FH at a young age are vastly missed in Germany. Thus, it appears that the single initiation letter that we issued was not sufficient to provide a broad awareness of the significance of LDL-C. Other reasons to waive cholesterol testing may be that it has been disputed that the cost-effectiveness of systematic risk factor screening in the absence of vascular disease is still a matter of debate^39^ or that lipid lowering therapy would not immediately have been considered in a given patient.

### Acceptance of FH ALERT

Amongst all participating physicians, 200 (14%) decided to opt out. We believe that this discontinuation rate is satisfactory since we offered a hitherto completely unknown service. As the most common reason to withdraw physicians quoted that the ALs produced too much additional paperwork and due to this often lead to lacking time in their practice to even read individual ALs. Other withdrawing physicians said that it would need too much time to follow the advice provided with the ALs, disregarding that a clear diagnosis could facilitate treatment decisions and patients’ adherence in the future and might therefore allow time savings alongside. Refinement of the FH ALERT strategy in the future will therefore have to resolve this criticism, for instance by cumulating results within one weekly report. However, of the physicians who finished the pilot study almost more than two thirds assessed the initiative as positive, and only less than 10% had a negative opinion about the FH ALERT project.

### Further diagnostic activity including genetic testing

The 462 physicians who received an alert ordered 93 sampling kits for genetic testing, of which 26 (28%) were ultimately returned for genetic testing. We recorded the number of genetic tests requested until 6.5 months after the conclusion of FH ALERT, but evidently the time between ordering kits and actually carrying out the test may be longer so that actual number of tests may yet be slightly higher.

Overall, the offer of genetic testing for FH was thus received with some caution, although the environment for cascade screening, which is recommended as cost-effective for identifying FH patients and preventing vascular disease (reviewed in Ref.^15^), would be ideal in a rural area like the Upper Palatinate where the general practitioner usually oversees many generations of a family. However, if one assumes that those 13 people who either received the diagnosis of genetic hypercholesterolemia or definite FH would gain 10 years of life each through intensive treatment, the FH ALERT pilot project would have saved 130 years of life and the effect would even be much larger if treating physicians went back to the families of the index patients to identify other affected individuals.

The reasons for the still cautious uptake of genetics may be manifold. Ordering genetic tests may have been uncommon in ambulatory care so far. It has even been argued that the clinical diagnosis of FH is good enough in terms of awareness. However, for many decades, the diagnosis of FH had been made by clinical criteria. This did not overcome the underdiagnosis of FH. Consequentially, lipid experts recommend that the diagnosis of FH is established by genetic means^15^. Other reasons may include: lack of knowledge of FH amongst physicians, misbeliefs about the expenses or the reimbursement of genetic testing (genetic testing is not charged to the laboratory budget of physicians in Germany), lack of time, lack of diligence, low awareness of FH in the lay population, poor willingness of patients to agree into genetic testing. It is, however, unlikely that the patients’ attitudes would seriously compromise the use of genetic testing. Rather parents of affected children or patients have been open to genetic testing recognizing its benefit with little evidence for psychological impact^42,43^. Consequently, we consider proactive and comprehensive education of physicians of utmost importance to lead them up to a better understanding of the benefits of genetic testing and the most recent recommendations. This is evidently not only true for genetic lipid disorders but applies to all disease areas in which genetic disease becomes symptomatic in adulthood.

We noted 101 new visitors on the www.fhscore.eu website. Under the assumption that there was an overlap between the 101 new visitors on the website and the 60 physicians who ordered kits for genetic testing, it may be conservative to assume that 120 physicians amongst the 462 who had received an AL responded in any of the two ways to the FH ALERT initiative. Thus, the overall response rate may be 25% or more. Since to our knowledge the design of FH ALERT is unprecedented, a comparison to established benchmarks is hard to accomplish. In any case, however, the response rates achieved here by far exceed the response rates known from untargeted mailings which are usually 1% or less.

### Diagnostic yield in relation to the potential number of FH patients in the target population

Of the 26 requests for genetic testing ten were inconspicuous, three showed variants most likely not related to hypercholesterolemia, eight samples showed variants or combinations of variants which we classified as polygenic and 5 (19%) showed at least one variant that we deemed causal for familial hypercholesterolemia. This yield may be less than obtained in specialized lipid clinics, where we found causal mutations in approximately 80% of patients with a DLCN Score of 6 or more^13^. Yet our diagnostic yield was still higher than in a recent Norwegian study, in which 29,449 unrelated persons suspicious of FH were genetically tested amongst which 2818 (9.6%) tested positive for mutations in the genes LDLR, APOB and PCSK9^44^.

In four out of five FH cases the interpretation of the genetic results is unambiguous. In one case it may be disputed. This patient carries the following mutations: ABCG5 c.1829A>C, p.(Glu610Ala); APOB c.2630c>T, p.(Pro877Leu). Both mutations have not been described in the literature and therefore had to be classified as class 3 formally. Because, however, both were classified as damaging in silico, we classified them as responsible for the phenotype of the patient.

There is substantial overlap in LDL-C levels among subjects with and without FH and many with LDL-C levels below the thresholds used in our study will also have FH. Therefore, molecular diagnostics has for good reasons been recommended to distinguish between these two groups. According to Khera et al.^4^, only less than 2% of subjects with and LDL-C ≥ 190 mg/dl in the general population have an FH-mutation. Assuming their screening approach and ours being similar, approximately 50 FH patients could have been diagnosed among our 2846 patients triggering an alert. Thus the 5 FH cases found make up approximately 10% of the number of patients theoretically expected in our study. This obviously represents a marked improvement over the general diagnoses rate of FH which has been estimated 1% for Germany^6^, and it had been achieved within a period as short as 3 months. If one would then still account for the cases in which we considered the genetic findings as polygenic hypercholesterolemia, this would increase to approximately 19%.

We used second generation sequencing of 14 candidate genes putatively implicated in FH. Limiting sequencing to the Loci LDLR, APOB and PCSK9 could have to be considered to allow for cheaper and simpler testing. However, the recent advances in sequencing technology brought the costs of sequencing broad panels down to the level of targeting a few genes with Sanger sequencing, thus providing significantly more information at the same speed and expense. For this reason, we went for the most recent approach, which is practically cost-neutral.

We also wish to place our approach into the context of what could realistically be expected: between 1994 and 2009, and thus within a period of 16 years, the Dutch Lipid Clinics Cascade Screening identified approximately 46,000 FH cases. These patients track back to approximately 5350 index patients (ratio of total FH patients to index cases by cascade screening 8.6:1^45^ in a target population of approximately 16,000,000 inhabitants). This means an annual rate of identification of index patients of approximately 2.23 per 100,000 or a monthly identification rate of approximately 0.19 index patients per 100,000 of whom roughly two third are provable by genetic means.

Our target population was 60,812 individuals (after accounting for repetitive testing, see below) and we identified 5 FH cases with genetic FH within 3 months. This corresponds to the identification of 2.74 index patients per month and 100,000 people. Thus, our approach seems to be approximately 14-fold more efficient than the Dutch Lipid Clinics Cascade Screening which is considered to most efficient approach to FH screening so far. Subsequent cascade screening of the families of the patients identified would still amplify the success of our program.

### Future directions

3512 ALs ultimately lead to 26 genetic tests. Despite this, we consider our pilot study successful, mainly because significant other diagnostic activity (visits to FH score website) developed during the project period. Also, the high proportion of physicians rating FH positive has been encouraging and we are convinced that our novel strategy of communication between referrers and the laboratory holds considerable potential for the future.

Based on our experience, conceivable improvements are:

i) reducing the amount of paperwork by consolidating individual alert letters to weekly cumulated alert letters;
ii) generating increased awareness for TC/LDL-C earlier before starting the initiative by educational events and multiple initiation letters;
iii) including medical societies and key opinion leaders in awareness activities;
iv) increasing the frequency of calls by the call center to remind physicians to perform genetic testing and send back the genetics kits for analyses;
v) yearly repetition of the initiative.

### Limitations

Limitations of this project are the exclusion of samples from patients above 60 years of age and the thresholds for ALs at the 95th percentile of the TC and LDL-C distribution. The latter obviously will miss some FH patients (also including the treated ones), because having an LDL-C of less than 190 mg/dl does not rule out FH^4^. Further, we did not include samples coming from hospitals to which FH patients may present with acute coronary syndromes. FH ALERT did not distinguish between patients with or without lipid-lowering treatment. FH Patients on treatment may have been missed by this approach. However, this bias seems to be presumably moderate, because patients at high risk also including FH patients are grossly undertreated, as it has been illustrated in recent research^46–49^.

The pilot study was restricted to 3 months only and to a rural area of Germany and may therefore not be representative. It is also a limitation that it was hard to place our findings into the context of similar studies because to the best of our knowledge there is no published report systematically evaluating our approach.

Finally, this is neither a clinical trial nor does our study allow to estimate the frequency of FH in the target population. Rather it is a piece of “real-world” implementation research shedding light on the hurdles still encountered in the identification of FH patients.

## Conclusions

We conclude that bridging the interface between referring physicians and the laboratory by an innovative approach of communication is technically demanding, but feasible. Our initiative was welcome and highly appreciated by the participating physicians. Although individualized recommendations as they were provided with FH ALERT were not rigorously translated into concrete action throughout, our strategy has proven more effective than conventional FH screening, represents a major step forward and should encourage future activities in the field.

## References

[1] Ference BA (2017). Low-density lipoproteins cause atherosclerotic cardiovascular disease. 1. Evidence from genetic, epidemiologic, and clinical studies. A consensus statement from the European Atherosclerosis Society Consensus Panel. Eur. Heart J..

[2] Klose G, Laufs U, März W, Windler E (2014). Familial hypercholesterolemia: developments in diagnosis and treatment. Deutsches Arzteblatt Int..

[3] Nordestgaard BG (2013). Familial hypercholesterolaemia is underdiagnosed and undertreated in the general population: Guidance for clinicians to prevent coronary heart disease: consensus statement of the European Atherosclerosis Society. Eur. Heart J..

[4] Khera AV (2016). Diagnostic yield and clinical utility of sequencing familial hypercholesterolemia genes in patients with severe hypercholesterolemia. J. Am. Coll. Cardiol..

[5] Sanna C (2016). Homozygous familial hypercholesterolemia in childhood: Genotype-phenotype description, established therapies and perspectives. Atherosclerosis.

[6] Schmidt N (2017). Familial hypercholesterolemia in primary care in Germany. Diabetes and cardiovascular risk evaluation: Targets and Essential Data for Commitment of Treatment (DETECT) study. Atherosclerosis.

[7] Beheshti SO, Madsen CM, Varbo A, Nordestgaard BG (2020). Worldwide prevalence of familial hypercholesterolemia: Meta-analyses of 11 million subjects. J. Am. Coll. Cardiol..

[8] Mundal L (2014). Mortality among patients with familial hypercholesterolemia: A registry-based study in Norway, 1992–2010. J. Am. Heart Assoc..

[9] Versmissen J (2008). Efficacy of statins in familial hypercholesterolaemia: A long term cohort study. BMJ.

[10] Williams RR (1993). Diagnosing heterozygous familial hypercholesterolemia using new practical criteria validated by molecular genetics. Am. J. Cardiol..

[11] Scientific Steering Committee on behalf of the Simon Broome Register Group (1991). Risk of fatal coronary heart disease in familial hypercholesterolaemia. BMJ.

[12] Defesche JC, Lansberg PJ, Umans-Eckenhausen MA, Kastelein JJ (2004). Advanced method for the identification of patients with inherited hypercholesterolemia. Semin. Vasc. Med..

[13] Grenkowitz T (2016). Clinical characterization and mutation spectrum of German patients with familial hypercholesterolemia. Atherosclerosis.

[14] Goldberg AC (2011). Familial hypercholesterolemia: Screening, diagnosis and management of pediatric and adult patients: Clinical guidance from the National Lipid Association Expert Panel on Familial Hypercholesterolemia. J. Clin. Lipidol..

[15] Sturm AC (2018). Clinical genetic testing for familial hypercholesterolemia: JACC Scientific Expert Panel. J. Am. Coll. Cardiol..

[16] Catapano AL (2016). 2016 ESC/EAS guidelines for the management of dyslipidaemias: The task force for the management of dyslipidaemias of the European Society of Cardiology (ESC) and European Atherosclerosis Society (EAS) Developed with the special contribution of the European Assocciation for Cardiovascular Prevention & Rehabilitation (EACPR). Atherosclerosis.

[17] Mach F (2019). ESC/EAS Guidelines for the management of dyslipidaemias: Lipid modification to reduce cardiovascular risk. Eur. Heart J..

[18] Allgemeinmedizin, D. G. f. Hausärztliche Risikoberatung zur kardiovaskulären Prävention. S3-Leitlinie. *AWMF-Register-Nr. 053-024, DEGAM-Leitlinie Nr. 19.* https://www.degam.de/files/Inhalte/Leitlinien-Inhalte/Dokumente/DEGAM-S3-Leitlinien/053-024_Risikoberatung%20kardiovaskul.%20Praevention/053-024l_Hausa%CC%88rztliche_Risikoberatung_kardivaskula%CC%88re_Praevention_29-08-2018.pdf (2017). Accessed on August 1, 2020.

[19] Richards S (2015). Standards and guidelines for the interpretation of sequence variants: A joint consensus recommendation of the American College of Medical Genetics and Genomics and the Association for Molecular Pathology. Genet. Med..

[20] Ballantyne CM, Vega GL, East C, Richards G, Grundy SM (1987). Low-density lipoprotein metabolism in cerebrotendinous xanthomatosis. Metabolism.

[21] Cohen JC (2006). Multiple rare variants in NPC1L1 associated with reduced sterol absorption and plasma low-density lipoprotein levels. Proc. Natl. Acad. Sci. USA.

[22] Wang LJ (2011). Molecular characterization of the NPC1L1 variants identified from cholesterol low absorbers. J. Biol. Chem..

[23] Bennet AM (2007). Association of apolipoprotein E genotypes with lipid levels and coronary risk. JAMA.

[24] Wang LR, McIntyre AD, Hegele RA (2018). Complex genetic architecture in severe hypobetalipoproteinemia. Lipids Health Dis..

[25] Argyri L (2014). Molecular basis for increased risk for late-onset Alzheimer disease due to the naturally occurring L28P mutation in apolipoprotein E4. J. Biol. Chem..

[26] Kamboh MI (1999). A novel mutation in the apolipoprotein E gene (APOE*4 Pittsburgh) is associated with the risk of late-onset Alzheimer's disease. Neurosci. Lett..

[27] Hansel B (2014). Premature atherosclerosis is not systematic in phytosterolemic patients: Severe hypercholesterolemia as a confounding factor in five subjects. Atherosclerosis.

[28] Buonuomo PS (2017). Timely diagnosis of sitosterolemia by next generation sequencing in two children with severe hypercholesterolemia. Atherosclerosis.

[29] Alves AC, Etxebarria A, Soutar AK, Martin C, Bourbon M (2014). Novel functional APOB mutations outside LDL-binding region causing familial hypercholesterolaemia. Hum. Mol. Genet..

[30] Motazacker MM (2013). Evidence of a polygenic origin of extreme high-density lipoprotein cholesterol levels. Arterioscler. Thromb. Vasc. Biol..

[31] Pirillo A (2017). Spectrum of mutations in Italian patients with familial hypercholesterolemia: New results from the LIPIGEN study. Atheroscler. Suppl..

[32] Dron JS, Hegele RA (2017). Complexity of mechanisms among human proprotein convertase subtilisin-kexin type 9 variants. Curr. Opin. Lipidol..

[33] Holla OL (2009). Effects of intronic mutations in the LDLR gene on pre-mRNA splicing: Comparison of wet-lab and bioinformatics analyses. Mol. Genet. Metab..

[34] Maxwell KN (2016). Evaluation of ACMG-guideline-based variant classification of cancer susceptibility and non-cancer-associated genes in families affected by breast cancer. Am. J. Hum. Genet..

[35] Marmontel O (2018). Single, short in-del, and copy number variations detection in monogenic dyslipidemia using a next-generation sequencing strategy. Clin. Genet..

[36] Bertolini S (2013). Spectrum of mutations and phenotypic expression in patients with autosomal dominant hypercholesterolemia identified in Italy. Atherosclerosis.

[37] Hobbs HH, Brown MS, Goldstein JL (1992). Molecular genetics of the LDL receptor gene in familial hypercholesterolemia. Hum. Mutat..

[38] Cassanelli S (1998). A 'de novo' point mutation of the low-density lipoprotein receptor gene in an Italian subject with primary hypercholesterolemia. Clin. Genet..

[39] Piepoli MF (2016). 2016 European Guidelines on cardiovascular disease prevention in clinical practice: The Sixth Joint Task Force of the European Society of Cardiology and Other Societies on Cardiovascular Disease Prevention in Clinical Practice (constituted by representatives of 10 societies and by invited experts) Developed with the special contribution of the European Association for Cardiovascular Prevention & Rehabilitation (EACPR). Eur. Heart J..

[40] Stone NJ (2013). ACC/AHA Guideline on the treatment of blood cholesterol to reduce atherosclerotic cardiovascular risk in adults: A report of the American College of Cardiology/American Heart Association Task Force on Practice Guidelines. Circulation.

[41] Grundy SM (2018). 2018 AHA/ACC/AACVPR/AAPA/ABC/ACPM/ADA/AGS/APhA/ASPC/NLA/PCNA guideline on the management of blood cholesterol. Circulation.

[42] Umans-Eckenhausen MA, Defesche JC, van Dam MJ, Kastelein JJ (2003). Long-term compliance with lipid-lowering medication after genetic screening for familial hypercholesterolemia. Arch. Intern. Med..

[43] Claassen L, Henneman L, van der Weijden T, Marteau TM, Timmermans DR (2012). Being at risk for cardiovascular disease: Perceptions and preventive behavior in people with and without a known genetic predisposition. Psychol. Health Med..

[44] Leren TP, Bogsrud MP (2021). Molecular genetic testing for autosomal dominant hypercholesterolemia in 29,449 Norwegian index patients and 14,230 relatives during the years 1993–2020. Atherosclerosis.

[45] Umans-Eckenhausen MA, Defesche JC, Sijbrands EJ, Scheerder RL, Kastelein JJ (2001). Review of first 5 years of screening for familial hypercholesterolaemia in the Netherlands. Lancet.

[46] Schmidt N (2017). CaRe high—Cascade screening and registry for high cholesterol in Germany. Atheroscler. Suppl..

[47] März W (2018). Utilization of lipid-modifying therapy and low-density lipoprotein cholesterol goal attainment in patients at high and very-high cardiovascular risk: Real-world evidence from Germany. Atherosclerosis.

[48] Fox KM (2018). Treatment patterns and low-density lipoprotein cholesterol (LDL-C) goal attainment among patients receiving high- or moderate-intensity statins. Clin. Res. Cardiol..

[49] Ray KK (2020). EU-wide cross-sectional observational study of lipid-modifying therapy use in secondary and primary care: The DA VINCI study. Eur. J. Prev. Cardiol..

